# Alterations in the expression of homologous recombination repair (HRR) genes in breast cancer tissues considering germline *BRCA1/2* mutation status

**DOI:** 10.1007/s10549-024-07441-4

**Published:** 2024-07-30

**Authors:** Izabela Laczmanska, Rafal Matkowski, Stanislaw Supplitt, Pawel Karpinski, Mariola Abrahamowska, Lukasz Laczmanski, Adam Maciejczyk, Ewelina Czykalko, Ewelina Iwaneczko, Piotr Kasprzak, Bartłomiej Szynglarewicz, Maria Sasiadek

**Affiliations:** 1 Department of Genetics, Faculty of Medicine, Wroclaw Medical University, Marcinkowskiego 1, 50-368 Wroclaw, Poland; 2 Lower Silesian Oncology, Pulmonology and Hematology Center, Hirszfeld Sq. 12, 53-413 Wroclaw, Poland; 3 Department of Oncology, Faculty of Medicine, Wroclaw Medical University, Hirszfeld Sq. 12, 53-413 Wroclaw, Poland; 4 Laboratory of Genomics and Bioinformatics, Hirszfeld Institute of Immunology and Experimental Therapy, Polish Academy of Sciences, Weigla 12, 53-114 Wroclaw, Poland

**Keywords:** Homologous recombination (HR), *BRCA1/2* deficiency, Real-time PCR, Breast cancer

## Abstract

**Introduction:**

Homologous recombination (HR) is a crucial DNA-repair mechanism, and its disruption can lead to the accumulation of mutations that initiate and promote cancer formation. The key HR genes, *BRCA1* and *BRCA2*, are particularly significant as their germline pathogenic variants are associated with a hereditary predisposition to breast and/or ovarian cancer.

**Materials and methods:**

The study was performed on 45 FFPE breast cancer tissues obtained from 24 and 21 patients, with and without the germline *BRCA1/2* mutation, respectively. The expression of 11 genes: *BRCA1, BRCA2, ATM, BARD1, FANCA, FANCB, FANCI, RAD50, RAD51D, BRIP1,* and *CHEK2* was assessed using Custom RT2 PCR Array (Qiagen), and results were analysed using R.

**Results:**

Cancer tissues from patients with *BRCA1* or *BRCA2* germline mutations displayed no significant differences in the expression of the selected HR genes compared to *BRCA1* or *BRCA2* wild-type cancer tissues. In *BRCA1*^mut^ cancer tissues, *BRCA1* expression was significantly higher than in *BRCA2*^mut^ and *BRCA* wild-type cancer tissues.

**Conclusions:**

In cancer tissues harbouring either *BRCA1* or *BRCA2* germline mutations, no significant differences in expression were observed at the mRNA level of any tested HR genes, except *BRCA1*. However, the significant differences observed in *BRCA1* expression between germline *BRCA1*^mut^, germline *BRCA2*^*mut*^ and *BRCA1/2*^wt^ tissues may indicate a compensatory mechanism at the mRNA level to mitigate the loss of *BRCA1* function in the cells.

## Introduction

Breast cancer (BC) is a highly heterogeneous disease with many different histological, molecular, and clinical subtypes. The molecular heterogeneity is observed at the genetic, epigenetic, and transcriptomic levels. This, combined with the high level of morbidity, makes BC an interesting subject of scientific studies focused on understanding the causes and mechanisms of BC development and progression [1–3].

Each BC is unique and differs at the molecular level. Thus, the classification of BC based on histological and immunohistochemical features does not reflect the complex genetic changes underlying tumour growth and development [4].

Gene expression analyses allow for identifying molecular BC subtypes, thus, enabling tailored therapy. Moreover, currently, available multigene prognostic panels, such as Oncotype Dx and MammaPrint, rely on the expression of various genes in the tumour tissue and, thus, differ in the range of prognostic and/or predictive capabilities. Therefore, developing new tests based on wide genetic analyses and introducing them into clinical practice are a promising area in the diagnosis and treatment of this cancer [5, 6].

Maintaining genetic stability is extremely difficult and is enabled by constantly active DNA-repair systems [7, 8]. An accumulation of DNA mutations resulting from ineffective DNA repair leads to genome instability, a key reason for cancer development and progression. Therefore, mutations in DNA-repair genes are frequent targets for tailored therapies [8, 9].

It has been revealed that in BC patients with *BRCA1/2* mutation, the effectiveness of poly(ADP-ribose) polymerase (PARP) inhibitors contributes to the extension of disease-free survival. PARP1, an enzyme involved in DNA repair, participates in the correction of DNA single-strand breaks (SSBs), which, if unrepaired, may convert into double-stranded DNA breaks (DSBs) during DNA replication. DNA replication-derived DSBs result in chromosome breakages and translocations, leading to severe genome instability [10]. The pivotal mechanism in repairing DSBs is the homologous recombination (HR) leading to the replacement of DSBs with an undamaged sequence [11–14]. Inactivating mutations in HR genes leads to the accumulation of other mutations in the genome, causing changes that promote carcinogenesis. The therapy of HR-deficient BC patients with PARP1 inhibitors (PARPi) causing synthetic lethality results in deleterious accumulation of mutations and, thereafter, apoptosis [15, 16]. Thus, PARP inhibitors are effective in the therapy of patients with *BRCA1/2*^mut^ breast cancer and homologous recombination deficiency (HRD) [5, 15, 16].

Many studies confirm that *BRCA*-dependent carcinogenesis is related to HR system inactivation [11, 17, 18]. Other HR genes involved in BC development include *PALB2*, *BARD1, TP53*, *BRIP1*, and *RAD51C* [19]. Previous research revealed that mutations in the above-mentioned genes significantly decrease DSB repair activity [20].

Our study aims to evaluate the alterations in the expression/transcript level of the following HR genes: *BRCA1, BRCA2, ATM, BARD1, FANCA, FANCB, FANCI, RAD50, RAD51D, BRIP1*, and *CHEK2*. As a result, we aim to develop a potential prognostic marker in patients diagnosed with *BRCA-*positive BC.

## Material

Breast cancer tissue samples were secured by the core needle biopsy before the patient's systemic treatment to assess the expression level of selected HR genes in tumour cells.

Core needle and vacuum-assisted breast biopsy (CNB and VABB) samples from 45 BC patients were collected and preserved in formalin-fixed paraffin-embedded (FFPE) blocks for subsequent RNA extraction and analysis. A pathologist selected the most representative BC tissue sections containing approximately 50% of cancer cells (macrodissection). All samples were taken before the chemotherapy. Later, all patients received treatment based on standard chemotherapy regimens (anthracyclines, taxanes).

In all patients, partial (PR) or complete pathological response (CR) was observed after neoadjuvant therapy (11 PR *vs* 33 CR). The complete pathological response was defined as the disappearance of all invasive cancer tissue in the resected breast specimen and in all sampled regional lymph nodes after the completion of neoadjuvant chemotherapy.

All patients were referred for a consultation with a clinical geneticist regarding hereditary breast and ovarian cancer predisposition. In the case of the germline *BRCA1/2* pathogenic or likely pathogenic variant, consultation with a clinical geneticist and the appropriate molecular test were also offered to the patient’s adult close relatives.

### Ethics statement

All patients signed informed consent. The study was approved by the Ethics Committee of Wroclaw Medical University (Nos. 611/2019 and 65/2023). All patients were diagnosed and treated in the Breast Unit, Lower Silesian Oncology, Pulmonology, and Hematology Center, Wroclaw, Poland. All samples were taken as part of the patient’s diagnostic and therapeutic scheme. All procedures performed in this study followed the principles for medical research of the 1964 Declaration of Helsinki and its later amendments or comparable ethical standards.

## Methods

The pathogenic *BRCA1* or *BRCA2* germinal variants were assessed from blood using NGS *BRCA1/2* panel testing (Devyser BRCA NGS CE-IVD, MiSeq Dx Illumina). Polish recurrent pathogenic variants were estimated/assessed using a laboratory-developed PCR screening test, whose results were confirmed by Sanger sequencing (BigDye™ Terminator v3.1 Cycle Sequencing Kit, ThermoFisher Scientific; Termocykler C1000 Touch Thermal Cycler, BioRad; ABI 3500 Series Genetic Analyzer, ThermoFisher Scientific).

### RNA extraction

Total RNA was extracted from breast cancer FFPE tissue sections using a Qiagen® RNeasy FFPE Mini Kit according to the manufacturer’s instructions. The RNA quality was determined using a NanoPhotometer N60 (Implen). The samples with an RNA concentration of ≥ 60 ng/µl, an A260/A280 ratio of 1.8 ~ 2.0, and an A260/A230 ratio of 2 ~ 2.2 were accepted for the analysis.

### PCR array

**Fig. 1:**
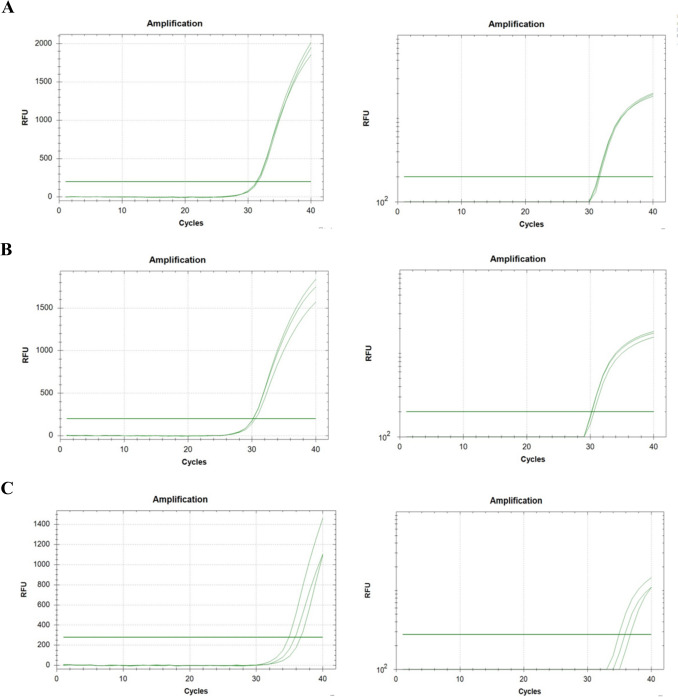
Representations of real-time PCR amplification curves. Three replicates of the amplification reaction for each sample are shown either on a linear scale (left) or on a semi-log scale (right). Figure 1**A** The plot for *BRCA1* mRNA expression in *BRCA1*-mutated tissue (Cq: 31.31/31.55/31.39); Fig. 1**B** The plot for *BRCA1* mRNA expression in *BRCA2*-mutated tissue (Cq: 30.15/30.19/30.52); Fig. 1**C** The plot for *BRCA1* mRNA expression in *BRCA1/2*-unmutated tissue (Cq: 36.65/34.89/35.82)

The Qiagen® Custom RT2 Profiler PCR Array was used to analyse the expression level of 11 HR genes (*BRCA1, BRCA2, ATM, BARD1, FANCA, FANCB, FANCI, RAD50, RAD51D, BRIP1,* and *CHEK2*) and two or three housekeeping genes (*GAPDH* and *B2M*, and for *BRCA1/2* additionally *ACTB*). All genes were analysed in triplicates with respective housekeeping genes and controls included in the PCR plate according to the producer protocol using the real‐time cycler Bio-Rad CFX96 (Fig. 1).

### Statistical analysis

Data analysis was performed using R/Bioconductor environment and HTqPCR and compareGroups packages [PMID: 19,808,880; https://www.jstatsoft.org/article/view/v057i12]. Raw data were normalised using the B2M gene as a housekeeping reference. Data distribution of continuous variables was assessed using the Shapiro–Wilks test. Depending on whether the variable was considered as continuously normal-distributed, continuous non-normal-distributed, or categorical t-test, the Kruskal–Wallis test or chi-squared test were used, respectively. The significance level was set at 0.05.

## Results

The study group consisted of 45 women aged 35–76 years (average age 53 years), including 24 women with a confirmed diagnosis of breast cancer and *BRCA1/2* germline mutation and 21 women diagnosed with invasive breast cancer and no *BRCA1/2* germline mutations.

All patients received systemic neoadjuvant therapy. The receptor status (ER, PR, and HER2), Ki67 status, adjuvant therapy response, and histopathological classification are presented in Table 1

**Table 1:** Clinical data of the study group (24 patients with *BRCA1*/*2* mutations)

No	Hist-pat	TNM before treatment	*BRCA1/2* variant	HER2-status [positive (P) / negative (N)]	ER-status [%]	PR-status [%]	Ki67-status [%]	CR/PR
1	infiltrating duct carcinoma, NOS G3	T2N1aM0	*BRCA1*:c.4689C>G	P	100	5	60	CR
2	infiltrating duct carcinoma, NOS G3	T1bN0M0	*BRCA1*:c.4986+4A>T	N	0	0	80	CR
3	infiltrating duct carcinoma, NOS G3	T1cN0M0	*BRCA1*:c.5509T >G	P	0	0	25	CR
4	infiltrating duct carcinoma, NOS G3	T2N0M0	*BRCA1*:c.4675+1G>A	N	0	5	40	CR
5	Mixed invasive ductal and lobular breast carcinoma	T1cN0M0	*BRCA1*:c.4689C>G	P	100	90	40	CR
6	infiltrating duct carcinoma, NOS G3	T2N0M0	*BRCA1*:c.4689C>G	P	0	0	50	CR
7	infiltrating duct carcinoma, NOS G3	T2N3M0	*BRCA1*:c.220C>T	N	0	0	90	CR
8	infiltrating duct carcinoma, NOS G3	T2N0M0	*BRCA1*:c.4357+1G>C	N	15	0	70	CR
9	infiltrating duct carcinoma, NOS G3	T1cN0M0	*BRCA1*:c.5266dup	N	0	0	90	CR
10	infiltrating duct carcinoma, NOS G3	T2N0M0	*BRCA1*:c.5266dup	N	0	0	50	PR
11	infiltrating duct carcinoma, NOS G3	T2N0M0	*BRCA1*:c.3700_3704del	N	0	0	90	CR
12	infiltrating duct carcinoma, NOS G3	T2N1M0	*BRCA1*:c.5266dup	P	0	1	90	CR
13	infiltrating duct carcinoma, NOS G3	T2N0M0	*BRCA1*:c.5266dup	P	30	0	80	CR
14	infiltrating duct carcinoma, NOS G3	T1cN0M0	*BRCA2*:c.6405_6409del	N	10	2	70	CR
15	infiltrating duct carcinoma, NOS G3	T2N1M0	*BRCA2*:c.7007G>A	P	100	70	25	CR
16	infiltrating duct carcinoma, NOS G2	T2N2M0	*BRCA2*:c.7680dup	P	10	0	30	PR
17	infiltrating duct carcinoma, NOS G2	T1cN0M0	*BRCA2*:c.4483_4484del	N	0	0	0	CR
18	infiltrating duct carcinoma, NOS G2	T2N1M0	*BRCA2*:c.1796_1800del	P	100	30	50	PR
19	infiltrating duct carcinoma, NOS ST G2	T2N1aM0	*BRCA2*:c.658_659del	N	90	10	25	CR
20	infiltrating duct carcinoma, NOS G2	T2N1M0	*BRCA2*:c.5238dup	P	100	100	30	CR
21	infiltrating duct carcinoma, NOS G3	T2N0M0	*BRCA2*:c.818C>G	N	100	80	30	CR
22	infiltrating duct carcinoma, NOS G2	T3N0M0	*BRCA2*: c.5205_5208del	P	100	15	40	PR
23	infiltrating duct carcinoma, NOS G2	T1cN2M0	*BRCA2*: c.3975_3978dup	N	100	40	40	CR
24	infiltrating duct carcinoma, NOS G3	T1N0M0	*BRCA2*:c.857C>G	P	20	0	60	CR
					AV 41%	AV 19%	AV 52%	

**Fig. 2:**
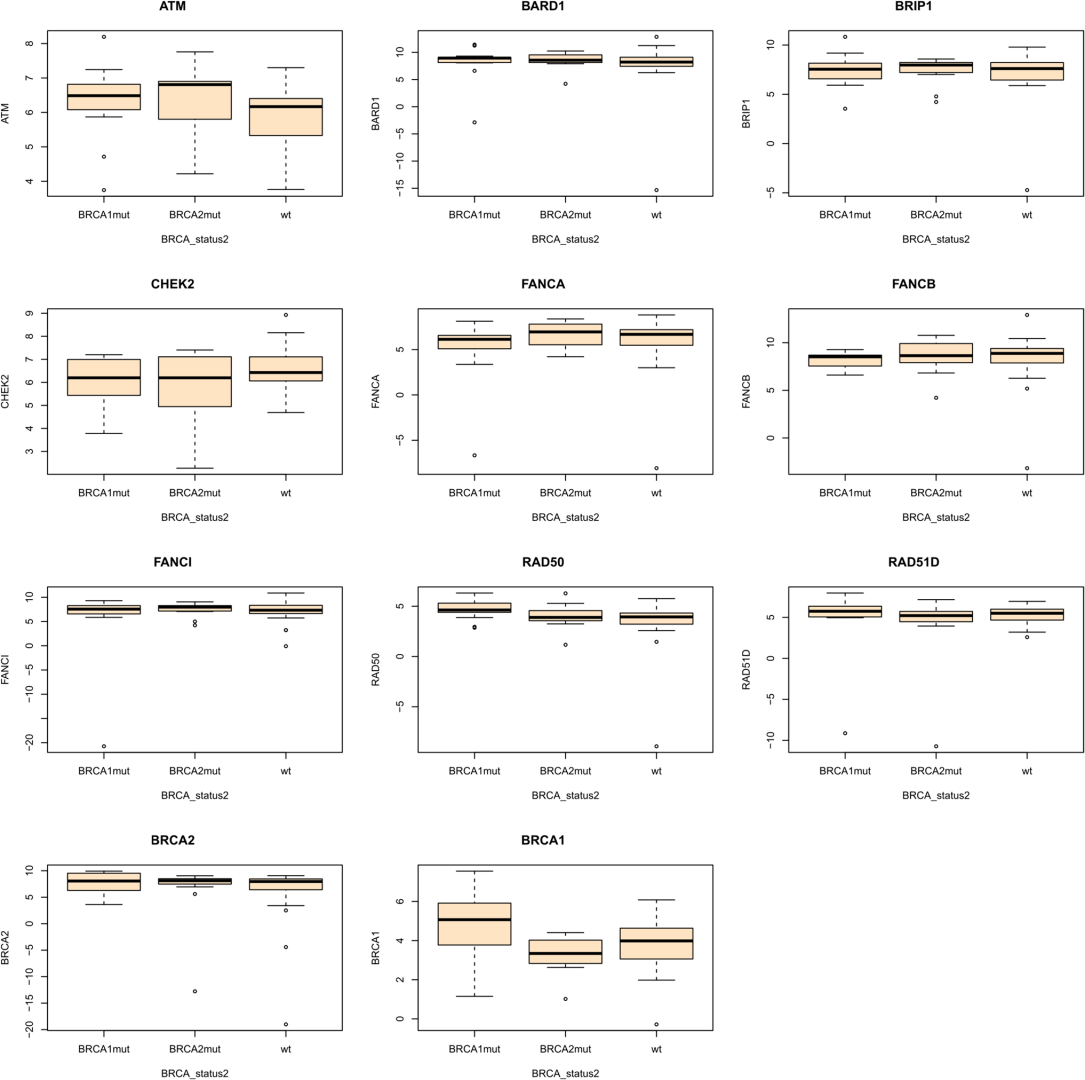
Box plots representing expression levels of selected HR genes considering germline *BRCA1/2* mutation status

No significant difference has been found for *ATM, BARD1, FANCA, FANCB, FANCI, RAD50, RAD51D, BRIP1,* and *CHEK2* expression in *BRCA1/2*^mut^ cancer tissues as compared to *BRCA1/2*^wt^ samples (Table 2, Fig. 2).

**Table 2:** Clinical data of the study group (21 patients without *BRCA1*/*2* mutations)

No	Hist-pat	TNM before treatment	*BRCA1/2* variant	HER2-status [positive (P) / negative (N)]	ER-status [%]	PR-status [%]	Ki67-status [%]	CR/PR
1	invasive lobular carcinoma, NOS G2	T3N3M1	no	P	100	50	50	PR
2	invasive lobular carcinoma, NOS G2	T1cN1aM0	no	P	90	90	7	CR
3	infiltrating duct carcinoma, NOS G2	T1cN0M0	no	P	100	100	20	CR
4	infiltrating duct carcinoma, NOS G2	T2N0M0	no	P	0	5	40	CR
5	infiltrating duct carcinoma, NOS G2	T1bcN0M0	no	P	60	0	10	CR
6	infiltrating duct carcinoma, NOS G2	T4N1M0	no	P	5	0	20	CR
7	infiltrating duct carcinoma, NOS G3	T2N0M0	no	P	0	0	80	PR
8	infiltrating duct carcinoma, NOS G2	T1cN0M0	no	P	90	90	30	CR
9	infiltrating duct carcinoma, NOS G3	T1cN0M0	no	P	100	0	30	PR
10	infiltrating duct carcinoma, NOS G2	T1cN1M0	no	P	100	10	20	PR
11	infiltrating duct carcinoma, NOS G2	T1cN0M0	no	P	100	20	30	CR
12	infiltrating duct carcinoma, NOS G2	T1cN1M0	no	N	0	0	60	CR
13	invasive lobular carcinoma, NOS G2	T1bN0M0	no	P	100	100	15	CR
14	infiltrating duct carcinoma, NOS G3	T2N0M0	no	P	100	20	30	CR
15	infiltrating duct carcinoma, NOS G2	T2N0M0	no	P	0	90	20	CR
16	invasive lobular carcinoma, NOS G2	T4bN2M0	no	N	100	100	20	PR
17	infiltrating duct carcinoma, NOS G2	T4bN2M0	no	N	100	70	50	PR
18	infiltrating duct carcinoma, NOS G2	T1cN0M0	no	N	0	0	50	CR
19	infiltrating duct carcinoma, NOS G2	T1bN0M0	no	P	100	7	15	CR
20	infiltrating duct carcinoma, NOS G3	T3N3M0	no	N	0	0	60	PR
21	invasive lobular carcinoma, NOS G2	T3N0M0	no	P	90	100	40	CR
					AV 64%	AV 46%	AV 33%	

^*^ Ki67 status: 5–7% low, 11–59% medium, 60–100 high; PR – partial, CR – complete pathological response

The only statistically significant difference was observed for the *BRCA1* gene expression. In *BRCA1*^mut^ cancer tissues, the *BRCA1* expression level was significantly higher than in *BRCA2*^mut^ and *BRCA1/2*^wt^ cancer tissues (Fig. 1). Apart from this observation, we found that *BRCA1*^mut^ cancer tissues displayed significantly higher expression of Ki67 and a significantly higher proportion of ER and PR negative cases. Moreover, patients with *BRCA1*^mut^ responded better to the applied therapy (higher proportion of complete response) than *BRCA2*^mut^ and/or *BRCA1/2*^wt^ patients (Table 3).

**Table 3:** Summary descriptives table by groups of *BRCA1/2* status

	[ALL]	*BRCA1*^mut^	*BRCA2*^mut^	*BRCA1/2*^wt^	p.overall
Number of cases (N)	*N* = 45	*N* = 13	*N* = 11	*N* = 21	
age	53.0 [43.2;64.0]	48.0 [43.2;55.2]	59.0 [39.0;64.5]	57.0 [47.0;68.0]	0.288
HER2_status:					0.360
negative	18 (40.0%)	7 (53.8%)	5 (45.5%)	6 (28.6%)	
positive	27 (60.0%)	6 (46.2%)	6 (54.5%)	15 (71.4%)	
Ki67_status:					0.008
high	12 (26.7%)	8 (61.5%)	2 (18.2%)	2 (9.52%)	
intermediate	31 (68.9%)	5 (38.5%)	8 (72.7%)	18 (85.7%)	
low	2 (4.44%)	0 (0.00%)	1 (9.09%)	1 (4.76%)	
Ki67	40.0 [25.0;60.0]	70.0 [50.0;90.0]	30.0 [27.5;45.0]	30.0 [20.0;40.0]	0.001
ER_status:					0.005
negative	15 (33.3%)	9 (69.2%)	1 (9.09%)	5 (23.8%)	
positive	30 (66.7%)	4 (30.8%)	10 (90.9%)	16 (76.2%)	
ER	90.0 [0.00;100]	0.00 [0.00;15.0]	100 [15.0;100]	100 [5.00;100]	0.009
PR_status:					0.047
negative	18 (40.0%)	9 (69.2%)	3 (27.3%)	6 (28.6%)	
positive	27 (60.0%)	4 (30.8%)	8 (72.7%)	15 (71.4%)	
PR	5.00 [0.00;70.0]	0.00 [0.00;1.00]	15.0 [1.00;55.0]	20.0 [0.00;90.0]	0.012
Therapy response:					0.233
CR	31 (72.1%)	11 (91.7%)	7 (63.6%)	13 (65.0%)	
PR	12 (27.9%)	1 (8.33%)	4 (36.4%)	7 (35.0%)	
*ATM*	6.30 [5.66;6.84]	6.49 [6.08;6.82]	6.81 [5.80;6.90]	6.17 [5.33;6.40]	0.284
*BARD1*	8.54 [7.97;9.27]	8.95 [8.13;9.06]	8.57 [8.12;9.54]	8.21 [7.43;9.11]	0.561
*BRIP1*	7.75 [6.57;8.22]	7.55 [6.57;8.16]	7.97 [7.21;8.23]	7.61 [6.45;8.22]	0.947
*CHEK2*	6.28 [5.56;6.99]	6.20 [5.44;6.99]	6.20 [4.95;7.11]	6.43 [6.06;7.11]	0.523
*FANCA*	6.56 [5.45;7.38]	6.13 [5.09;6.56]	6.95 [5.53;7.81]	6.68 [5.48;7.19]	0.232
*FANCB*	8.55 [7.84;9.36]	8.52 [7.55;8.70]	8.64 [7.90;9.91]	8.88 [7.87;9.39]	0.457
*FANCI*	7.56 [6.64;8.33]	7.56 [6.57;8.28]	7.97 [7.16;8.29]	7.34 [6.64;8.34]	0.827
*RAD50*	4.23 [3.38;4.69]	4.62 [4.39;5.31]	3.89 [3.56;4.56]	3.94 [3.22;4.33]	0.068
*RAD51D*	5.43 [4.86;6.07]	5.75 [5.05;6.36]	5.21 [4.47;5.72]	5.50 [4.67;6.00]	0.399
*BRCA2*	8.08 [6.41;8.58]	8.06 [6.28;9.53]	8.12 [7.48;8.50]	7.96 [6.41;8.45]	0.675
*BRCA1*	3.98 [3.06;4.94]	5.07 [3.77;5.91]	3.34 [2.83;4.02]	3.98 [3.06;4.63]	0.028

^*^ Ki67 status: 5–7% low, 11–59% medium, 60–100 high; *PR*–partial, *CR*–complete pathological response

## Discussion

Transcriptome profiling may be a powerful and effective method for searching for new molecular biomarkers of cancer prognosis, risk of progression, cancer-free survival, and other clinical features. Recent studies confirmed that a high homologous recombination deficiency (HRD) score is associated with poor survival among BC, prostate cancer, glioma, and head and neck squamous cell carcinoma (HNSCC) patients [21–25]. Hence, HRD, resulting from the loss of function of HR genes, including *BRCA1/2*, has been approved as an independent predictive biomarker of sensitivity to PARPi therapy [15, 16, 26]. Moreover, recent studies suggest a potential predictive value of HRD status in platinum-based chemotherapy in breast [27] and ovarian cancer (OC) patients [28].

Our study assessed the expression of the selected main HR pathway genes in cancer tissue to check whether their mRNA levels are altered in the *BRCA1/2*^mut^ BC. The rationale for this research was based on the extensive interconnections among HR proteins, where deregulation of a single but critical gene’s expression could potentially impact the expression of others.

The only statistically significant differences were observed for *BRCA1*, as its mRNA level was elevated in *BRCA1*-mutated tissues compared to *BRCA2*-mutated and *BRCA1/2* wild-type tissues.

Our results are consistent with those published by Wang et al., who proved that *BRCA1* and *BRCA2* gene expression is upregulated in breast and ovarian cancer (OC) tissues. Moreover, these authors observed an increased expression of *NF1* and *SYCP2* genes, interacting with *BRCA1/2* genes in the regulation of the cell cycle. Therefore, they suggested the importance of functional interrelations among the *BRCA1/2* with the other genes involved in BC and OC development and progression, thus, influencing the clinical course of disease and treatment outcomes [29].

In their recent study on 38 ovarian cancer *vs* 11 fallopian tube tissues, Custódio et al. showed that *BRCA1/2* mRNA expression varied between individual samples. Moreover, in tissues characterised by downregulated *BRCA1/2* expression, the other 12 genes involved in the HR pathway also exhibited low mRNA levels. The analysis of 299 ovarian cancer samples from The Cancer Genome Atlas (TCGA) confirmed these findings [30]. It is important to emphasise that our study’s findings on *BRCA*^mut^ breast cancer tissues differ, as we observed elevated mRNA levels for *BRCA1*. This discrepancy may arise from the varying schemes and methodologies employed in the studies: 1) the cancer type (BC vs OC) or 2) different HR genes studied (*ERCC1, ERCC2, ERCC3, ERCC4, ERCC5, ERCC6, ERCC8, EXO1, FAN1, FANCA, FANCB, FANCC vs ATM, BARD1, FANCA, FANCB, FANCI, RAD50, RAD51D, BRIP1, CHEK2*), 3) different *BRCA1/2* mutational status (17/40 (42.5%) samples with *BRCA1/2* pathogenic or likely pathogenic variant *vs* 24/45 (53%)), and finally, the most importantly 4) different laboratory methods (very sensitive and accurate NanoString Technology and droplet digital PCR (ddPCR) *vs* real-time PCR).

Another study on 96 fresh frozen ovarian cancer tissues obtained from chemotherapy-naïve patients and patients after neoadjuvant chemotherapy was performed using a tailored NanoString-based Pancancer Pathway Panel of 19 HR genes and showed a correlation between over-expression of *C11osf30, NBN, FANCF, FANCC, FANCB, RAD50* and improved outcome in chemotherapy-naïve patients. Moreover, a correlation has been observed between over-expression of *BRCA2, TP53, FANCB,* and *RAD51* and worse outcomes in chemotherapy-treated patients. When adding the extent of debulking as a covariate, the expression of *NBN, FANCF*, *RAD50,* and *RAD51* was significant, respectively [31].

Also, in the bladder cancer cell lines, the expression of four DNA damage repair genes, including two MR genes, was evaluated respectively before and after chemotherapy. The authors of this study revealed that the increase of *BRCA1* and *RBBP8* expression induced by chemotherapy correlates with worse sensitivity to treatment in non-basal and non-luminal cell lines. In contrast, no significant differences in the basal-cell lines most sensitive to chemo and radiotherapy were observed. This observation revealed the high diversity of HR genes expression, which correlates with the histopathological characteristics of tumours [32]. Moreover, in 413 bladder cancer samples (data derived from TCGA), a significantly higher expression level of four genes, *RAD21, RAD51, BARD1,* and *ERBB2,* was observed in *ERBB*-low as compared to *ERBB*-high tumours. Also, the combined expression of two out of four tested genes has been shown to correlate with chosen clinical features. This confirms the interconnections among the expression of different HR pathway genes [33].

In sporadic gastric cancer patients after receiving postoperative adjuvant chemotherapy, *BRCA1/BRCA2* expression assessed using IHC and mRNA tests displayed a correlation between *BRCA2*-elevated expression with advanced tumour stage but not disease-free and overall survival [34].

These findings indicate that the investigation should encompass not only the alterations in individual HR genes but also their interrelations, considering the clinical course of the disease alongside histopathological and biochemical variables characterising the studied cancer.

## Conclusion

The mRNA expression of *BRCA1* is upregulated in breast cancer tissues harbouring *BRCA1* mutation. However, no relevant differences in the expression of other HR genes have been observed between *BRCA1/BRCA2*^mut^ and non-mutated BC tissues. Hence, it appears that *BRCA*^mut^ tissues do not exhibit crucial compensatory alterations in the mRNA expression of other HR genes.

## Limitations

The prognostic value of HR gene expression in BC could not be assessed due to the limited sample size and the variability in histopathological classifications.

## Data Availability

No datasets were generated or analysed during the current study.
