# Correction: Alterations in the expression of homologous recombination repair (HRR) genes in breast cancer tissues considering germline *BRCA1/2* mutation status

**DOI:** 10.1007/s10549-024-07544-y

**Published:** 2024-11-23

**Authors:** Izabela Laczmanska, Rafal Matkowski, Stanislaw Supplitt, Pawel Karpinski, Mariola Abrahamowska, Lukasz Laczmanski, Adam Maciejczyk, Ewelina Czykalko, Ewelina Iwaneczko, Piotr Kasprzak, Bartłomiej Szynglarewicz, Maria Sasiadek

**Affiliations:** 1 Department of Genetics, Faculty of Medicine, Wroclaw Medical University, Marcinkowskiego 1, 50-368 Wroclaw, Poland; 2 Lower Silesian Oncology, Pulmonology and Hematology Center, Hirszfeld Sq. 12, 53-413 Wroclaw, Poland; 3 Department of Oncology, Faculty of Medicine, Wroclaw Medical University, Hirszfeld Sq. 12, 53-413 Wroclaw, Poland; 4 Laboratory of Genomics and Bioinformatics, Hirszfeld Institute of Immunology and Experimental Therapy, Polish Academy of Sciences, Weigla 12, 53-114 Wroclaw, Poland

**Correction to: Breast Cancer Research and Treatment (2024) 208:501–510** 10.1007/s10549-024-07441-4

In the original version of this article, the given and family names of the authors were incorrectly structured. The correct names should be Izabela Laczmanska, Rafal Matkowski, Stanislaw Supplitt, Pawel Karpinski, Mariola Abrahamowska, Lukasz Laczmanski, Adam Maciejczyk, Ewelina Czykalko, Ewelina Iwaneczko, Piotr Kasprzak, Bartłomiej Szynglarewicz, and Maria Sasiadek.

The original article has been corrected.

